# Eco-friendly degreasing adsorbent derived from oily scum and walnut shells for oilfield sewage treatment and industrial oils adsorption

**DOI:** 10.1371/journal.pone.0324631

**Published:** 2025-06-13

**Authors:** Chao Tang, Jiaojiao Guan, Yuliang Lin

**Affiliations:** 1 Faculty of Hydraulic Engineering, Chongqing Water Resources and Electric Engineering College, Yongchuan, Chongqing, China; 2 State Key Laboratory of Petroleum and Pollution Control, China National Petroleum Corporation, Beijing, China; Universiti Teknologi Petronas: Universiti Teknologi PETRONAS, MALAYSIA

## Abstract

Oily scum (OS) and walnut shells (WS) were employed to produce an eco-friendly degreasing adsorbent (DA) for the treatment of oilfield sewage and the adsorption of industrial oils. The optimal conditions for preparing DA were established as follows: a mass ratio of OS to WS of 1:2, a pyrolysis temperature of 600°C, a heating rate of 10°C/min, and a pyrolysis duration of 2 hours. The carbon mass fraction in DA was determined to be 90.72%, with a BET specific surface area of 563.31 m^2^/g, a pore volume of 0.794 cm^3^/g, and an average pore size of 5.618 nm. The pores were predominantly mesoporous, centered around a size of 3 nm. In oilfield sewage treatment tests, DA achieved a remarkable reduction of 92.64% in the petroleum content of oilfield sewage, outperforming activated carbon (AC) treatment by 13.52% under identical conditions. In tests for industrial oil adsorption, DA demonstrated initial adsorption rates of 138.55 mg/(g·min) for diesel and 189.52 mg/(g·min) for crude oil, achieving cumulative adsorption capacities of 973.3 mg/g and 1017 mg/g for diesel and crude oil, respectively. Thermal regeneration of DA significantly enhanced its oil affinity, markedly improving its initial adsorption rates. Furthermore, the cumulative adsorption capacity of regenerated DA for diesel and crude oil reached 966.88 mg/g and 946.15 mg/g respectively, surpassing that of regenerated AC for diesel (585.47 mg/g) and crude oil (849.21 mg/g). Additionally, DA showed a shorter penetration adsorption time, positioning it as a feasible option for recycling and as an eco-friendly emergency adsorbent in the context of industrial oil spills.

## Introduction

In the global effort to achieve carbon neutrality and mitigate climate change, managing oily sludge has become a critical issue within the oil and gas industry. Due to its hazardous properties, several countries have classified oily sludge as hazardous waste [1–4]. Pyrolysis technology can efficiently transforms the organic content of oily sludge into combustible gas, pyrolysis oil, and residual carbon [5–10], facilitating effective resource and energy recovery. Consequently, pyrolysis is regarded as a zero-waste approach for treating oily sludge [11,12].

A growing body of research indicates that increasing the organic components in sludge improves the quality of the products derived from its pyrolysis [13,14]. When external organic components are introduced that can alter sludge decomposition kinetics and thermodynamics, this adjustment typically results in the production of more desirable pyrolysis products [15–18]. For instance, during the production of adsorbent materials using oily sludge pyrolysis, the incorporation of biomass notably boosts both the yield and the adsorption capabilities of these materials [19–22]. Jun Wang et al. [23] synthesized micro-mesoporous enriched activated carbons (ACs) from mixtures of oily sludge and rice husk, achieving a higher specific surface area of 2575 m^2^/g compared to those prepared from the individual components alone (1100 m^2^/g and 1953 m^2^/g). Wang et al. [24] created aqueous phase sulfamethoxazole (SMZ) adsorption materials from combinations of various biomass materials with carbon-enriched oily sludge, employing pyrolysis technology. The resulting adsorption materials exhibited a maximum BET surface area of 1342 m^2^/g and an SMZ adsorption capacity of up to 361.9 mg/g. Han et al. [25] developed carbon materials from oily sludge and corn stalks through pyrolysis, applying them to Cr^6+^ removal. achieving a Cr^6+^ removal efficiency of 99.14%. The main objective of this research was to prepared an eco-friendly degreasing adsorbent (DA) from oily scum (OS) and walnut shell (WS). The deoiling properties of DA were investigated by evaluating its adsorption capabilities for petroleum in oilfield sewage, as well as its ability to adsorbed industrial oils, aiming to foster innovative utilization of waste resources in oil and gas fields, minimize the expenses associated with OS and WS disposal, and offer eco-friendly adsorption materials for the efficient treatment of oilfield sewage and the swift adsorption and disposal of industrial oil spills.

## Materials and methods

### Materials

OS was sourced from a sewage treatment plant in Liaohe Oilfield, with water, oil, and residue contents of 83.5%, 8.9%, and 7.6%, respectively. WS utilized in this study were supplied by Shijiazhuang Baori Environmental Technology Co., Ltd. These WS were dried in an oven at a temperature of 105 °C for 24 hours. Subsequently, they underwent pulverization and sieving to achieve a uniform particle size of 50 mesh. Elemental analysis conducted on the WS revealed a composition comprising 44.12% carbon (C), 6.28% hydrogen (H), 44.82% oxygen (O), 0.67% nitrogen (N), and 0.18% sulfur (S). Activated Carbon (AC) was procured from Dongguan Hongsheng Activated Carbon Co., Ltd., featuring a maximum water content of 10% and an ash content not exceeding 5%. The oilfield sewage utilized in this study was collected from the Shuguang oil production plant of Liaohe Oilfield. Before experimentation, the sewage was filtered through filter paper to remove suspended solids. Industrial oils, specifically diesel and crude oil, were obtained from Jingzhou Tianhe Technology Co., Ltd.

### Preparation method of DA

Refer to the preparation method of sludge-based adsorbent [26,27], OS and WS were thoroughly mixed at a mass ratio of 1:2 and loaded into a tubular furnace for pyrolysis. The pyrolysis process was conducted under a N_2_ atmosphere to ensure an inert environment. Upon completion of the pyrolysis reaction, the resultant solid material was extensively washed to dissolve all ash and oxides. After washing, the material was dried and ground to a particle size of 50 mesh to produce the DA.

### Analytical and test methods

The content of heavy metals and elemental composition were analyzed using an ICAP RQ ICP-MS and a Quantax 200X Flash 5000–10 EDS, respectively. Surface morphology and properties were examined using a Quanta 250 tungsten filament scanning electron microscope and NOVA-2000e N_2_ adsorption ASAP, respectively. The iodine value was determined in accordance with China’s national standard GB/T 7702.7-2023. The petroleum content in oilfield sewage was measured according to the national standard HJ 970–2018.

### Method of adsorbing petroleum in oilfield sewage

The static adsorption method employing ACs was utilized to investigate the adsorption capacity of DA for petroleum in oilfield sewage [28]. Two grams of DA were added to a conical flask containing 100 mL of oilfield sewage. The flask was subsequently placed in a water bath equipped with a constant temperature vibrator set at 28°C for one hour. Following the adsorption process, the mixture was filtered to separate the filtrate. The petroleum content in the filtrate was quantified, and the petroleum removal rate was calculated. To compare the efficacy, a parallel experiment was conducted using AC under identical conditions. The initial petroleum content in the oilfield sewage was determined to be 56.62 mg/L.

### Method of adsorbing industrial oils

**Fig 1:**
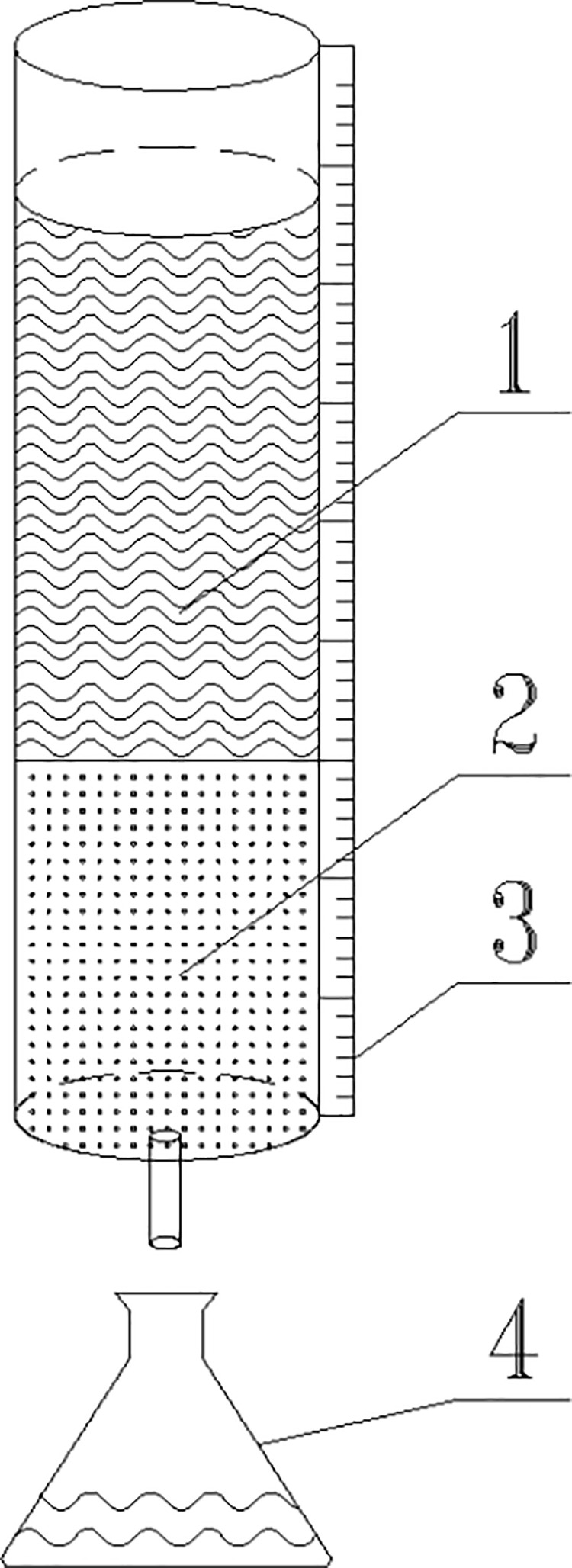
Oil adsorption device. **(1) Oil; (2) Adsorbent; (3) Tick mark; (4) Collection bottle**.

Following the dynamic adsorption test method for ACs [29], adsorption tests for industrial oils using adsorbents were conducted in plexiglass columns with an inner diameter of 40 mm and a length of 1000 mm. The industrial oils tested included crude oil and diesel. The experimental setup is depicted in Fig 1. During the test, 200 g of the adsorbent was loaded into the column, followed by a rapid injection of 400 mL of oil. The timing commenced immediately upon oil injection. The descent of the oil level was continuously recorded until the oil exited the column. Through volume-to-mass conversion, the cumulative oil adsorption capacity of the adsorbent was determined for various stages of the adsorption process. Subsequently, the adsorption rate of the adsorbent at different time intervals was calculated.

### Regeneration method of adsorbent

The adsorbent, post-adsorption, was subjected to pyrolysis in a tubular furnace at 600°C for one hour. Upon cooling, the regenerated adsorbent was obtained.

## Results and discussion

### Heavy metal content of OS

China’s national standards, such as the “Integrated Wastewater Discharge Standard” (GB 8978−1996), “Identification Standard for Hazardous Wastes—Identification for Extraction Toxicity” (GB 5085.3−2007), and “Agricultural Sludge Pollutant Control Standard” (GB 4284−2018), establish limits for the concentration of heavy metals in oily sludge. Table 1 displays the results of the analysis of heavy metal content in the leachate of OS, which indicate that the concentrations of heavy metals in the OS leachate are significantly below the maximum allowable emission concentrations specified by GB 8978−1996, GB 5085.3−2007, and the pollutant concentration limits for grade A sludge as outlined in GB 4284−2018. These findings confirm that the resource utilization process for OS does not pose a risk of secondary pollution from heavy metals.

**Table 1:** Heavy metal content in leachate of OS (w, μg/g).

Type	Heavy Metal Content							
	Cr6+	Hg	Ni	Cu	Zn	Cd	Pb	As
**OS**	0.17	/	0.01	0.13	0.10	/	0.08	0.03
**Ⅰ**	0.5	0.05	1.0	0.5	2.0	0.1	1.0	0.5
**Ⅱ**	5.0	0.1	5.0	100	100	1.0	5.0	5.0
**Ⅲ**	2.0	3.0	100	500	1200	3.0	300	30

**Ⅰ.** Maximum allowable emission concentration of “Integrated Wastewater Discharge Standard” (GB 8978−1996)

**Ⅱ.** Maximum allowable emission concentration of “Identification Standard for Hazardous Wastes-Identification for Extraction Toxicity” (GB5085.3−2007)

**Ⅲ.** Pollutant concentration limits for grade A sludge of “Agricultural Sludge Pollutant Control Standard” (GB 4284−2018)

### Preparation conditions of DA

The key factors influencing the preparation of DA include the mass ratio of OS to WS, pyrolysis temperature, pyrolysis heating rate, and pyrolysis holding time [21,30,31]. This study employed an orthogonal design with four factors and three levels (L9 (3^4^)) to ascertain the optimal conditions for DA preparation. The four factors were the mass ratio of OS to WS, pyrolysis temperature, pyrolysis heating rate, and pyrolysis holding time. Within the three levels, the mass ratio of OS to WS were 1:2, 1:1 and 2:1, pyrolysis temperature were 500°C, 600°C and 700°C, pyrolysis heating rate were 5 °C/min, 10 °C/min and 15 °C/min, pyrolysis holding time were 1h, 2h and 3h. The adsorption properties of DA were assessed using the iodine value under varying conditions. The orthogonal scheme and the results are presented in Table 2. The findings indicated that for factor A (the mass ratio of OS to WS), k_1_ (342.103) > k_2_ (323.853) > k_3_ (319.087), so A_1_ was the optimal level of factor A. Similarly, for factor B(pyrolysis temperature), k_2_ (359.563) was the largest, so B_2_ became the optimal level of Factor B. For factor C (Pyrolysis heating rate) and factor D (Pyrolysis holding time), C_2_ and D_2_ were the optimal levels, respectively. Therefore, the optimal configurations for these factors were determined to be A_1_, B_2_, C_2_, and D_2_. The ideal conditions for preparing DA were established as follows: a mass ratio of OS to WS of 1:2, a pyrolysis temperature of 600°C, a pyrolysis heating rate of 10 °C/min, and a pyrolysis holding time of 2 hours. Analysis of Range (R) values can ascertain the influence of each factor on the experimental outcomes. Upon comparing the R values of each factor, it was observed that R_B_ (63.036)> R_D_ (57.603)> R_A_ (23.016) > R_C_ (11.850). Which displayed that pyrolysis temperature exerted the most prominent effect on the preparation of DA, followed by pyrolysis holding time and mass ratio of OS to WS, pyrolysis heating rate had the least significant impact. The predominant influence of pyrolysis temperature arises from its direct control over three critical aspects: (1) the structural configuration of DA carbon skeleton, (2) the nucleation and evolutionary dynamics of pore architectures, and (3) the modulation of surface functional groups through thermal decomposition/rearrangement mechanisms. Furthermore, it governs the synergistic interactions during co-pyrolysis of OS and WS. As the predominant governing parameter in DA preparation, pyrolysis temperature dictates the thermodynamic equilibria and kinetic pathways of carbonization processes. In contrast, pyrolysis heating rate primarily regulates transient intermediate reactions along the thermal degradation pathway, while exerting minimal structural alterations on the final DA’s porous network. This fundamental disparity in mechanism-scale interaction explains why pyrolysis heating rate demonstrates the least significant impact on adsorption performance metrics [15,21,32,33].

**Table 2:** Orthogonal protocol and results table.

Number	Four factors and three levels				Iodine value, mg/g
**A (The mass ratio of OS to WS)**	**B (Pyrolysis temperature, °C)**	**C (Pyrolysis heating rate,°C/min)**	**D (Pyrolysis holding time, h)**
**1**	1:2	500	5	1	268.68
**2**	1:2	600	10	2	401.17
**3**	1:2	700	15	3	356.46
**4**	1:1	500	10	3	313.39
**5**	1:1	600	15	1	317.71
**6**	1:1	700	5	2	340.46
**7**	2:1	500	15	2	307.51
**8**	2:1	600	5	3	359.81
**9**	2:1	700	10	1	289.94
**k**_**1**_	342.103	296.527	322.983	292.110	
**k**_**2**_	323.853	359.563	334.833	349.713	
**k**_**3**_	319.087	328.953	327.227	343.220	
**R value**	23.016	63.036	11.850	57.603	
**Order**	B ＞ D ＞ A ＞ C				
**Optimal level**	A_1_	B_2_	C_2_	D_2_	
**Optimization**	A_1_B_2_C_2_D_2_				

### Characterization of materials

**Fig 2:**
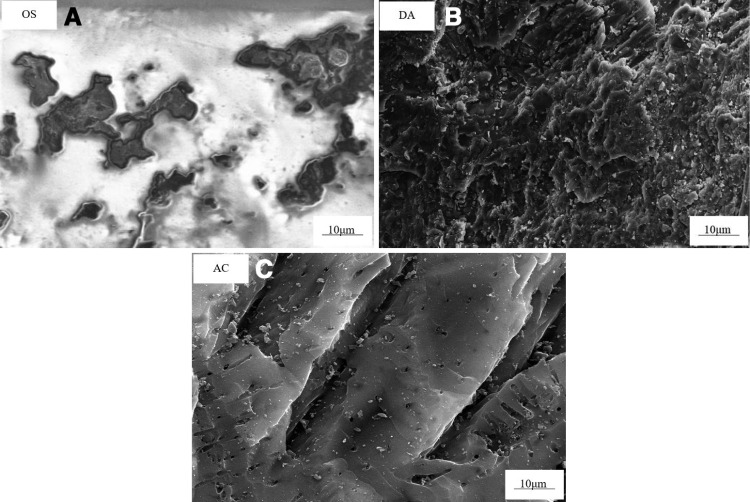
SEM images of OS, DA, and AC.

**Fig 3:**
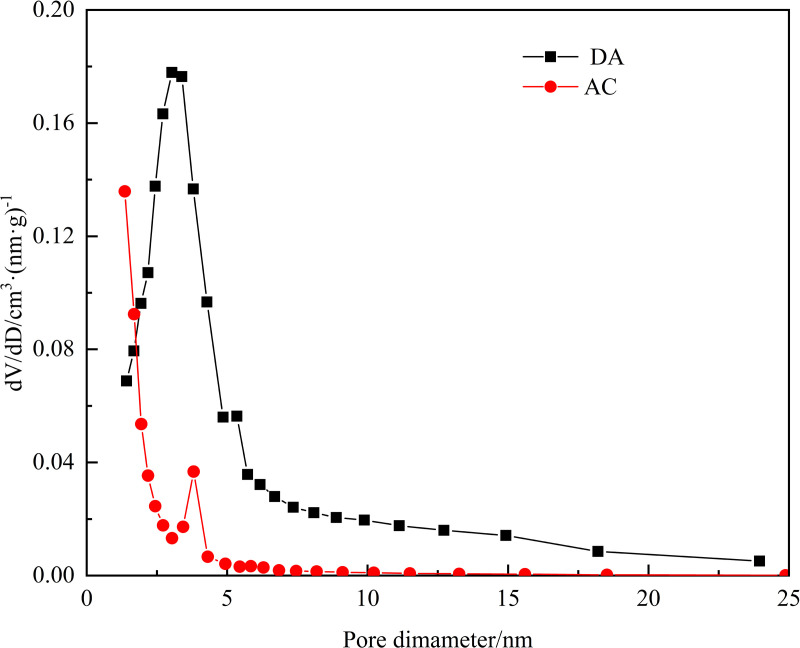
Pore-distribution curve of DA and AC.

Fig 2 displays the scanning electron microscopy (SEM) images of OS, DA, and AC, providing valuable insights into their respective surface characteristics. The surface of OS is characterized by a flat and smooth texture with a visible presence of oil. In contrast, DA exhibits a rough texture featuring numerous irregular pores and cracks of varying sizes. AC, however, presents a tightly packed structure with smaller pores and an even distribution. Fig 3 illustrates the pore size distribution of DA and AC, highlighting their distinct characteristics. The pore size distribution of AC is relatively concentrated, predominantly consisting of micropores around 1 nm. Conversely, DA’s pore size is primarily concentrated around 3 nm, with a distribution dominated by mesopores, indicating a considerably wider range. Wang et al. observed that AC prepared by co-processing oily sludge with rice husk exhibited reduced microporosity and more developed mesopores compared to carbon derived from oily sludge alone. They attributed this structural difference to the encapsulation of rice husk ash within the carbon skeleton formed by asphaltenes from the sludge, which promoted pore enlargement and suppressed micropore formation [23]. In the present study, WS likely fulfills a role analogous to that of rice husk in Wang’s system during the preparation of DA.

Table 3 summarizes the elemental compositions and surface characteristics of DA and AC. Both materials display comparable elemental profiles, with carbon constituting over 90% of their mass. This aligns with the typical carbon-rich nature of oily sludge-derived adsorbents [19,23–25,30]. For instance, Tang et al. reported a carbon content of 89.95% in waste tire-sludge adsorbent (WTSA) produced through co-pyrolysis [21]. Similarly, Mohammadi et al. demonstrated that KOH-activated pyrolysis of oil sludge yields porous carbon exceeding 80% carbon content, albeit with trace metallic constituents (Cd, Cu, Zn, Mn, Fe). Notably, these metals exhibited leaching rates below permissible thresholds in distilled water [34]. Surface characterization reveals distinct structural differences: DA possesses a Brunauer-Emmett-Teller (BET) specific surface area of 563.31 m^2^/g, substantially lower than that of AC and markedly inferior to both WTSA and AC-S1-3 derived from co-pyrolysis of oily sludge and rice husk [21,23]. However, DA compensates with larger pore volume and significantly greater average pore compared to AC. This inverse relationship between pore dimensions and surface area underscores the fundamental divergence in adsorption mechanisms between the two materials.

**Table 3:** Elemental composition and surface characteristic data of DA and AC.

Sample	DA	AC
**Elemental composition (W), %**		
**C**	90.72	91.03
**O**	6.57	7.18
**Na**	0.89	/
**Al**	0.29	0.85
**S**	1.05	0.61
**Si**	0.41	0.33
**BET Specific Surface Area, m** ^**2**^ **/g**	563.31	941.20
**Pore Volume, cm** ^**3**^ **/g**	0.794	0.501
**Average Pore Size, nm**	5.618	2.129

### Adsorption of petroleum in oilfield sewage by adsorbents

The adsorption performance of DA for petroleum in oilfield sewage was evaluated and compared with AC. As shown in Table 4, DA demonstrates superior petroleum adsorption capacity relative to AC. This enhanced performance correlates with DA’s significantly higher mesopore ratio and greater total pore volume. The well-developed mesoporous architecture provides an optimized environment for accommodating macromolecular organic compounds, enabling efficient migration and storage of petroleum within the adsorbent’s internal structure. This observation aligns with Wang et al.’s conclusion that mesopores play a critical role in adsorbent efficacy by facilitating the penetration of high-molecular-weight pollutants into pore networks. For instance, the SMZ adsorption material WO-2 derived from biomass-oily sludge co-carbonization exhibited lower specific surface area than its counterpart W-2, yet achieved higher adsorption capacity due to its broader pore size distribution and enhanced mesopore volume [24]. Similarly, Han et al. reported that mesopore-dominated biochar (OS-CS AC) demonstrated exceptional Cr(VI) removal efficiency [25], while Tang et al.’s WTSA showed analogous structural advantages [21].

**Table 4:** Comparison of DA and AC on Adsorption Effects of petroleum in Oilfield Sewage.

Sample	Petroleum Content before Adsorption, mg/L	Petroleum Content after Adsorption, mg/L	Petroleum Removal Rate, %
**DA**	56.62	4.17	92.64
**AC**		11.82	79.12
**Regenerated DA**		7.83	86.17
**Regenerated AC**		19.61	65.37

In contrast, AC’s microporous framework and restricted pore volume limit its effectiveness for macromolecular contaminant adsorption. These structural constraints impede both the diffusion kinetics and ultimate adsorption capacity for petroleum in oilfield sewage [24,35], resulting in AC’s comparatively inferior performance. Notably, both DA and AC exhibited diminished adsorption capacities after thermal regeneration, likely due to pore structure degradation. High-temperature regeneration processes may induce pore sintering through overactivation, leading to pore shrinkage and collapse of the hierarchical pore architecture, thereby reducing accessible adsorption sites.

### Adsorption of industrial oils by adsorbents

#### Adsorption rate.

**Fig 4:**
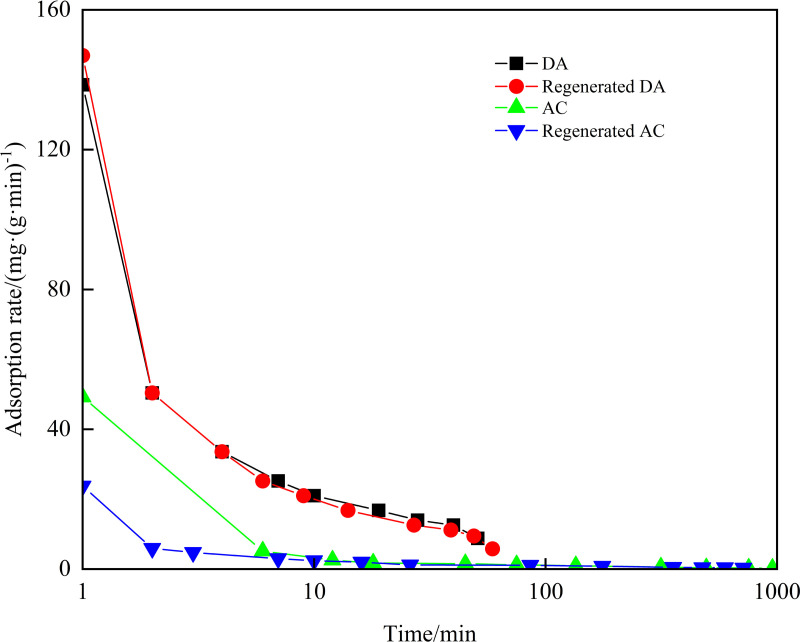
Adsorption rates of DA, AC and its regenerator on diesel.

**Fig 5:**
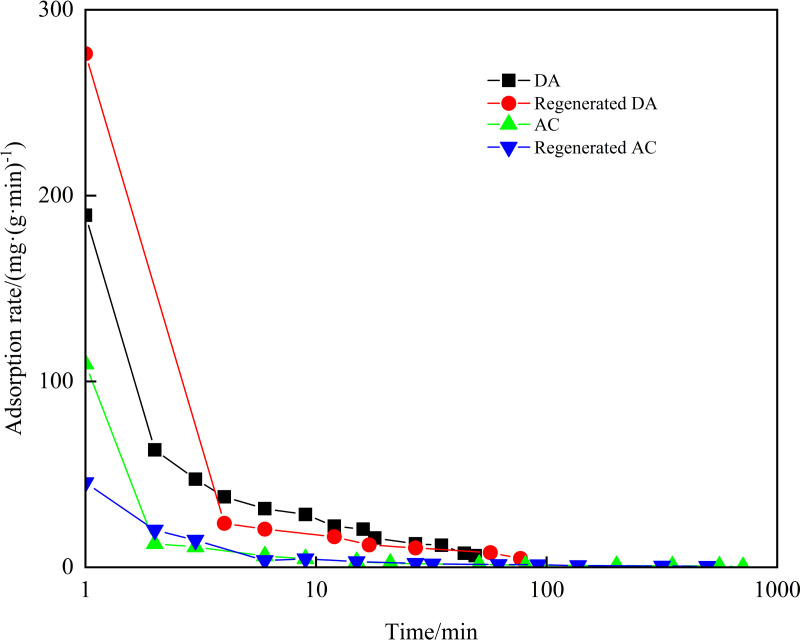
Adsorption rates of DA, AC and its regenerator on crude oil.

The adsorption rates of DA, AC and its regenerated agent on diesel and crude oil were investigated, with the results displayed in Figs 4 and 5. For diesel, the initial adsorption rate of regenerated DA was the fastest at an adsorption time of 1 minute, reaching 146.94 mg/(g·min), surpassing that of DA, which was 138.55 mg/(g·min). For crude oil, the initial adsorption rate of regenerated DA was also the fastest at 1 minute, achieving 276.39 mg/(g·min), significantly higher than that of DA, which was 189.52 mg/(g·min). This suggests that the thermal regeneration treatment enhanced the surface lipophilicity of DA, resulting in stronger oil adsorption and a higher initial adsorption rate under capillary action [36,37].Comparative analysis revealed DA’s superior adsorption rates over AC for both oil types. Specifically, DA demonstrated adsorption rates of 138.55 mg/(g·min) for diesel and 189.52 mg/(g·min) for crude oil, substantially higher than AC’s corresponding rates of 61.53 mg/(g·min) and 145.79 mg/(g·min). This performance disparity aligns with previous findings by Navarathna et al., who attributed enhanced oil adsorption in modified biochars to increased lipophilicity and hydrophobicity [38]. In this study, DA’s intrinsic properties as an oil sludge-derived adsorbent naturally confer strong lipophilicity and hydrophobicity, enabling faster oil capture than AC [39]. This mechanistic behavior is consistent with the industrial oil adsorption characteristics of WTSA [21].

#### Cumulative adsorption capacity.

**Fig 6:**
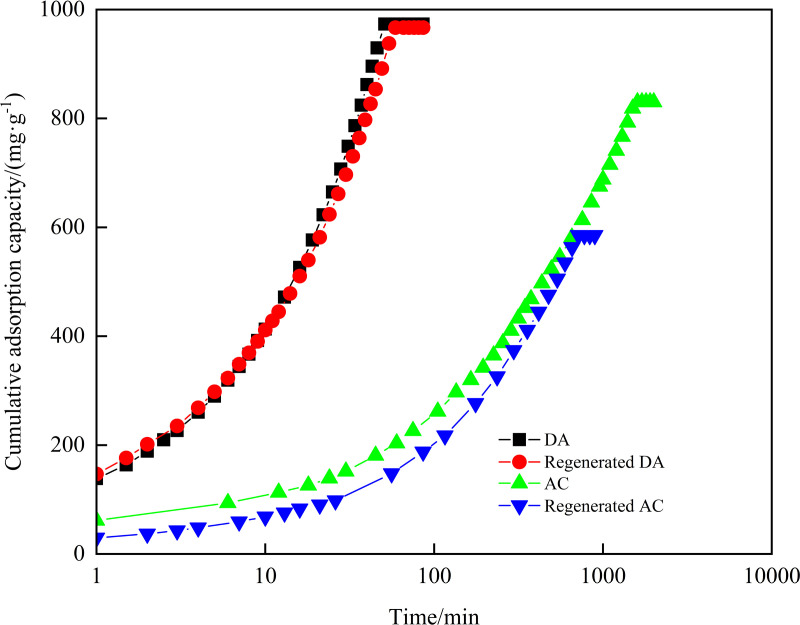
Cumulative adsorption capacity for diesel of DA, AC and its regenerator.

**Fig 7:**
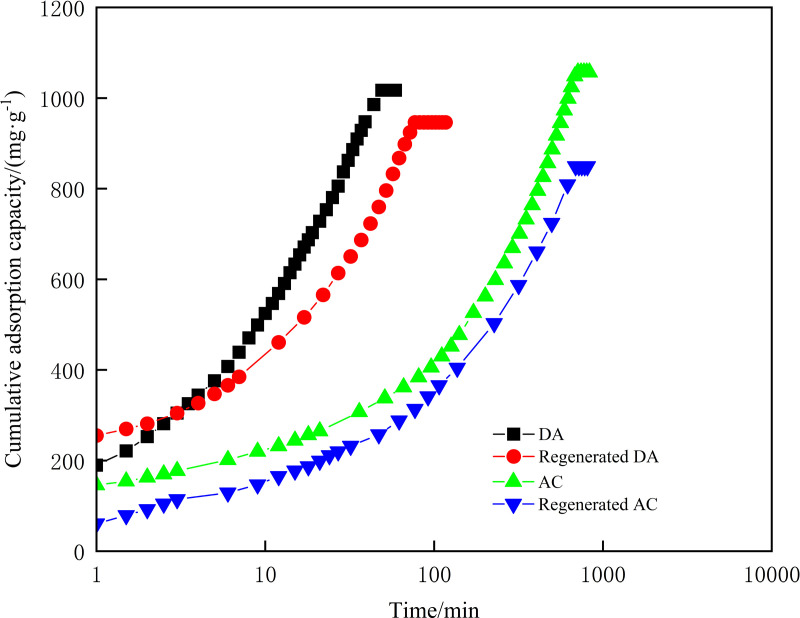
Cumulative adsorption capacity for crude oil of DA, AC and its regenerator.

The cumulative adsorption capacity of DA, AC and its regenerated agent on diesel and crude oil were evaluated, with the results detailed in Figs 6 and 7. It was observed that diesel passed through the adsorption bed of the column at 51 minutes for DA and 59 minutes for regenerated DA, with cumulative adsorption capacities of 973.3 mg/g and 966.9 mg/g, respectively. Similarly, crude oil passed through the adsorption bed at 49 minutes for DA and 77 minutes for regenerated DA, yielding cumulative adsorption capacities of 1017 mg/g and 946.2 mg/g, respectively. These results demonstrate that thermal regeneration preserved DA’s adsorption capacity without significant reduction. This observation aligns with findings by P. Márquez et al., who reported that thermal regeneration of granular AC in wastewater treatment effectively restores adsorption performance at appropriate temperatures, offering a cost-efficient alternative to nitrogen-atmosphere pyrolysis for AC reuse [36]. The stable regeneration efficiency of DA further supports its viability for post-adsorption recovery, underscoring its sustainability potential.

When comparing the cumulative adsorption capacity of DA and AC on industrial oils, it is evident that the cumulative adsorption capacity of AC for industrial oils was comparable to that of DA (with AC’s cumulative adsorption capacity being 830 mg/g for diesel and 1056 mg/g for crude oil). However, AC’s adsorption time for industrial oils through the adsorption bed of the adsorption column was significantly longer, with the penetration time for crude oil exceeded 700 minutes and for diesel surpassing 1600 minutes. This disparity corroborates the findings of R. Kandanelli et al., who emphasized that hydrophobic-lipophilic biochar materials facilitate rapid oil absorption [40]. The superior performance of DA for adsorption of industrial oils at the same adsorption duration is similar to that of WTSA [21], attributable to its enhanced mesoporous structure and pronounced lipophilic-hydrophobic properties [25,38].

## Conclusions

Eco-friendly degreasing adsorbent DA was synthesized via pyrolysis, with optimal conditions established as OS/WS mass ratio 1:2, pyrolysis temperature 600 °C, heating rate 10 °C/min, and 2-hour holding time. DA featured high carbon content and a predominant mesoporous structure centered at 3 nm. Compared to AC, DA demonstrated superior petroleum adsorption performance in oilfield sewage and a faster adsorption rate for industrial oils. At equivalent contact times, DA consistently achieved higher cumulative adsorption capacities for industrial oils than AC. The material also exhibited excellent thermal regeneration stability, enabling effective recovery and reuse after oils absorption.

## Supporting information

S1 Fig Adsorption of industrial oils by adsorbents. (DOCX)

S1 Table Orthogonal factor level table L9 (3^4^). (DOCX)
