# Supplementary figures and images for: Eco-friendly degreasing adsorbent derived from oily scum and walnut shells for oilfield sewage treatment and industrial oils adsorption
Source: PLoS One. 2025 Jun 13;20(6):e0324631. doi: 10.1371/journal.pone.0324631 (PMC12165379; doi:10.1371/journal.pone.0324631)

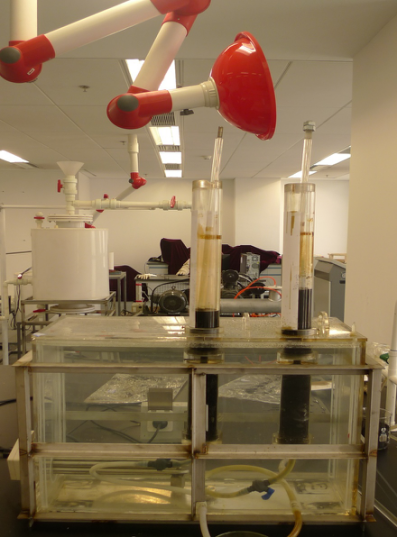


S1 Fig. Adsorption of industrial oils by adsorbents

Supplement: S1 Fig — (DOCX) [file pone.0324631.s001.docx]
