# Supplementary material for: Eco-friendly degreasing adsorbent derived from oily scum and walnut shells for oilfield sewage treatment and industrial oils adsorption
Source: PLoS One. 2025 Jun 13;20(6):e0324631. doi: 10.1371/journal.pone.0324631 (PMC12165379; doi:10.1371/journal.pone.0324631)
Supplement: S1 Table — (DOCX) [file pone.0324631.s002.docx]

**S2 Table. Orthogonal factor level table L9 (3^4^)**

| **Level** | **Factor A** | **Factor B** | **Factor C** | **Factor D** |
| --- | --- | --- | --- | --- |
|  | **The mass ratio of OS to WS** | **Pyrolysis temperature, ℃** | **Pyrolysis heating rate ,℃/min** | **Pyrolysis holding time, h** |
| **1** | 1:2 | 500 | 5 | 1 |
| **2** | 1:1 | 600 | 10 | 2 |
| **3** | 2:1 | 700 | 15 | 3 |
